# Influence of Salt Type on the Physicochemical, Microbiological, Sensory Properties, Mineral, Free Fatty Acid, and Volatile Composition of PDO Erzincan Tulum Cheese

**DOI:** 10.3390/foods15152665

**Published:** 2026-07-29

**Authors:** Ayla Arslaner, Özlem Yılmaz

**Affiliations:** 1 Department of Food Engineering, Faculty of Engineering, Bayburt University, Bayburt 69000, Türkiye; 2 Department of Hotel, Restaurant and Catering Services, Vocational School of Social Sciences, Bayburt University, Bayburt 69000, Türkiye

**Keywords:** PDO, Erzincan Tulum cheese, salt, low sodium, potassium, fatty acid, volatiles

## Abstract

This study evaluated the quality attributes of Protected Designation of Origin (PDO) Erzincan Tulum cheese by comparing control cheeses made with Kemah spring salt (KS) to those made with Sivas spring salt (SS), Çankırı rock salt (CS), and a low-sodium salt (rock salt–KCl blend, 50:50, LNaS). Cheeses were ripened at −2 to 0 °C for 120 days. Analyses included chemical composition, mineral profile, proteolysis, lipolysis, fatty acid composition, volatile compounds, and sensory properties. During ripening, dry matter and pH increased significantly (*p* < 0.05). Mineral content varied by salt type: LNaS had higher potassium and lower sodium, KS-C had the highest sodium and phosphorus, and SS-C the highest magnesium (*p* < 0.05). Proteolysis increased in all samples, with the highest levels in LNaS (*p* < 0.05). Lipolysis increased in all samples (*p* < 0.05), with CS-C showing the highest free fatty acid (FFA) content. Fifty volatile compounds were identified; carbonyl compounds were highest in LNaS-C and esters were highest in SS-C. Sensory evaluation showed that CS-C had the highest overall acceptability score and SS-C the lowest (*p* < 0.05). CS-C outperformed the control in sensory quality, and LNaS-C achieved similar sensory acceptance without compromising quality. Salt content and type were identified as critical factors in determining the authenticity of PDO Erzincan Tulum cheese.

## 1. Introduction

Cheese is a fermented dairy product with high nutritional value, and its production involves the interrelated preservation methods of fermentation, drying, and salting [1]. Among these preservation methods, sodium chloride (NaCl) is an essential ingredient in cheese production and many processed foods due to its important role in flavor development, texture formation, microbial safety, and food preservation [1,2]. The sodium chloride content of cheese ranges from 0.7% to 6.0%. This depends on the type of cheese and the salting method.

Cheese flavor is influenced by several factors, including milk type, processing conditions, salt and fat content, microbiota, and ripening, all of which affect proteolysis and lipolysis and the formation of characteristic aroma compounds [3,4,5]. Salt also regulates water activity, microbial growth, whey removal, texture development, and the ripening process, thereby playing a critical role in cheese quality [1]. Although salt is indispensable for cheese production, cheese is also an important dietary source of sodium, and excessive sodium intake has been associated with hypertension and cardiovascular diseases [6,7]. Consequently, the World Health Organization (WHO) recommends limiting sodium intake and encourages the use of low-sodium salt alternatives as a cost-effective strategy to reduce diet-related health risks [8]. Therefore, reducing sodium levels in processed foods such as cheese has become an important research priority.

Erzincan Tulum cheese is characterized by its white-to-cream color, high-fat content, semi-hard texture, and homogeneity. It has held Protected Designation of Origin (PDO) status in the European Union since 2024. It is influenced by the plant, animal, and microbiological biodiversity of its production area. The cheese is made from the milk of Akkaraman sheep that graze on plateaus in Erzincan province at altitudes above 1500 m. The cheese reflects local production expertise and supports regional economic development with its unique features [3,9]. Various natural and rock salts are used in the production of traditional Tulum cheeses in Türkiye. However, in cheeses subject to geographical registration other than Erzincan Tulum cheese, the source of the natural salt is not emphasized. One key feature of Erzincan Tulum cheese production is the use of Kemah spring salt for salting. Goatskin (Tulum) is often used as the packaging material. According to the PDO specification, Erzincan Tulum cheese must be ripened for at least 120 days [3,10].

Consistent with the recommendations of health authorities, the increasing demand from consumers and food producers to reduce salt content in processed foods has heightened interest in research focused on sodium reduction. One common practice in cheese production is the partial or complete replacement of sodium chloride (NaCl) with potassium chloride (KCl), calcium chloride (CaCl_2_), or magnesium chloride (MgCl_2_) [11,12,13]. However, salt substitution can result in notable changes in the sensory properties, texture, microbial stability, and ripening process of cheese [1]. Therefore, producing low-sodium cheese while maintaining safety and quality characteristics across different cheese varieties is essential. The naturalness of food is a key purchase motivation for many consumers, who often closely associate ‘natural’ with ‘healthy’. Accordingly, it is important that salt substitutes do not compromise the product’s perceived naturalness.

Previous research has addressed both reducing salt intake and partially or completely substituting sodium chloride with alternative salts. The effects of salt reduction on cheese quality vary by cheese variety. For instance, reducing salt in Cheddar cheese has been shown to negatively impact flavor and texture and to increase proteolysis [14]. In contrast, Mozzarella cheese retains its quality characteristics even with significant salt reduction [15]. These results suggest that salt-reduction strategies should be tailored to each cheese variety, as a universal approach is unlikely to be effective. The substitution of sodium chloride (NaCl) with potassium chloride (KCl) has been investigated in several cheeses, including Kefalograviera [16], Minas fresh cheese [17], Halloumi [18], São João [19], Mozzarella [20], Cheddar [21], Feta [12], and Kashar [7]. Currently, no studies have examined replacing sodium chloride with potassium chloride or using various natural-source salts in Tulum cheese production. In Anatolia, Çankırı rock salt and natural spring salts from the Sivas and Erzincan regions are the primary natural salts used in the processing of various fermented products, particularly cheeses. The present study evaluates physicochemical, microbiological, proteolytic water-soluble nitrogen (WSN), ripening index (RI), lipolytic (FFA%), fatty acid composition, volatile compounds, and sensory properties of Erzincan Tulum cheeses produced with low-sodium salt and different natural-source salts and compares them with the control cheese produced with traditional Kemah salt. Given the critical role of salt in defining the characteristic features of traditional cheeses such as Tulum cheese, it is necessary to assess the effects of sodium reduction and the use of alternative natural-source salts on product quality. Determining the quality characteristics of Tulum cheeses is important for both consumer health and the development of traditional products.

## 2. Materials and Methods

Raw ewe’s milk and rennet were acquired from nomads involved in sheep breeding on the Heybeli plateau in Erzincan province. Goatskin bags and Kemah spring salt were supplied by Tulum cheese producers in Erzincan. Çankırı rock salt and Sivas spring salt were purchased from local markets in Sivas. A low-sodium blend of natural rock salt and potassium chloride (KCl) (50:50, LNaS) was sourced from Estuz Food Industry and Trade Inc., Türkiye. The physicochemical and microbiological properties of the raw materials used for cheese production are presented in Table 1.

### 2.1. Experimental Design

Four cheese groups differing in salt type were produced in two independent trials. The sample labels, sample descriptions, and salt treatments are presented in Table 2. All cheeses were manufactured according to the traditional production method of Erzincan Tulum cheese, except for the salt type used. Cheeses were ripened in traditional tulums at −2 to 0 °C for 120 days. Each cheese sample was homogenized by thorough mixing, and representative subsamples were randomly collected for the planned analyses. Physicochemical, microbiological, and fatty acid analyses were performed on days 1 and 120 of ripening, whereas mineral composition, volatile compounds, and sensory evaluation were conducted at the end of ripening (day 120). Each analysis was performed using four analytical replicates.

### 2.2. Cheese Production Procedure

**Figure 1:**
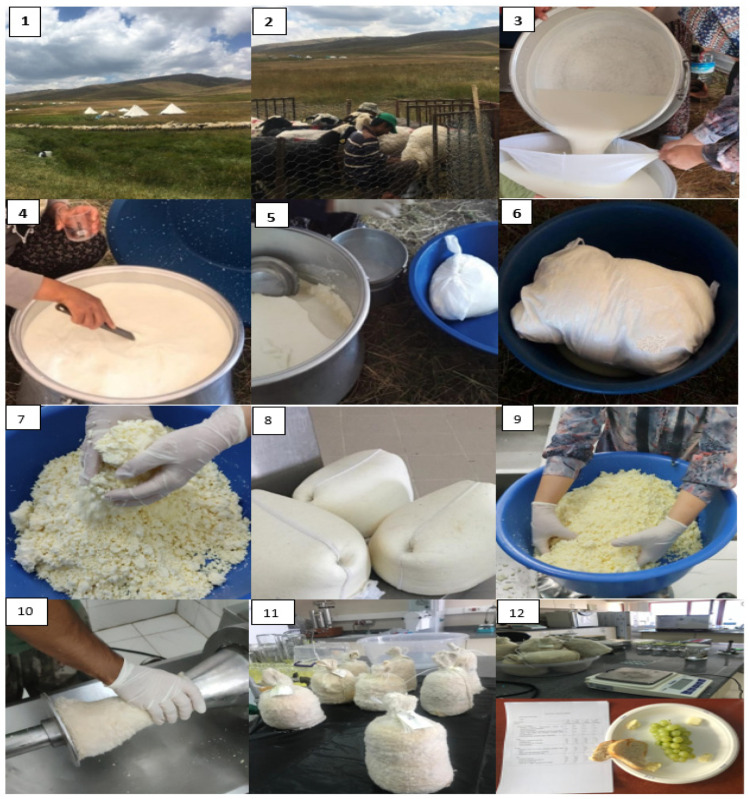
Production stages of Erzincan Tulum Cheese. (**1**–**6**) Plateau view; sheep milking; milk straining; rennet addition and coagulation within 90 min; coagulum cutting and draining for approximately 24 h; filling into bags and pre-ripening for 10 days at 8–10 °C. In the factory: (**7**–**10**) Curds divided into four equal parts and salted (KS, SS, CS, LNaS); second ripening at 18–20 °C for 10 days; cheese broken up; ground curd filled into goatskin (Tulum); final ripening for 120 days at 0–2 °C. Analyses (**11**,**12**).

The cheese samples were produced from Akkaraman ewe’s milk, following the traditional manufacturing procedure specified in the PDO specification [10]. Production took place in July 2024 at the tents of nomad families on the Heybeli plateau in Erzincan province, Türkiye. The process included: (1) hand-milking and filtering raw milk several times; (2) resting the milk cauldrons for about 2 h at 20 ± 1 °C; (3) coagulating the milk within 90 min by adding rennet; (4) cutting the coagulum and transferring the curd into cheesecloth-lined cauldrons to drain without pressing for 24 h; (5) the next day, manually breaking the drained cheese into smaller pieces and transferring it to 10 kg cloth bags. The first pre-ripening phase (10–11 days) occurred at the Heybeli plateau (2.200 m above sea level). (6) The cheese was divided into four equal portions, dry salted with 2% Kemah natural spring salt (KS), Sivas natural spring salt (SS), Çankırı natural rock salt (CS), or low-sodium salt (LNaS). (7) The second pre-ripening phase took place over 10 days at 18–20 °C at Tadın Food in Erzincan (8) Finally, samples (KS-C, SS-C, CS-C, and LNaS-C) were placed in goatskin containers for the final stage, ripening for 120 days at −2 to 0 °C as shown in Figure 1. A total of eight cheese samples, each weighing approximately 1 kg, were produced for each group (KS-C, SS-C, CS-C, LNaS-C). Four samples from each group were analyzed on day 1, while the remaining four were analyzed on day 120. The entire cheese-making process was performed in duplicate.

### 2.3. Physical and Chemical Analysis

The pH was measured using a Mettler Toledo pH meter. Total solids were determined gravimetrically. Salt content was determined by colorimetric titration, and water activity was measured using a Novasina LabMaster-aw instrument (Novasina AG, Lachen, Switzerland). Total nitrogen (TN) was determined by the Kjeldahl method [22]. The cheese ripening index (RI) was calculated as the percentage of WSN (water-soluble nitrogen) to TN. The WSN was determined according to the method of Butikofer et al. [23]. The fat content was determined using the butyrometric method [24]. 

### 2.4. Mineral and Heavy Metal Content Analysis

Multi-element determination was performed according to the method described by Ataro et al. [25]. Three replicates of each dried sample, each weighing approximately 0.5 g, were weighed into cleaned 50 mL Pyrex culture tubes. Each tube received 2 mL of concentrated, redistilled nitric acid (HNO_3_), covered with cling film, and was left overnight at room temperature for cold digestion. Tubes were then heated in a digestion block at 120 °C. As the liquid evaporated, an additional 2 mL of HNO_3_ was added, and the mixture was heated to dryness. This step was repeated until no reddish-brown (nitrogen dioxide) fumes remained and the solution was clear. Next, 1 mL of a 50:50 mixture of nitric acid and perchloric acid was added to each tube. The digestion block temperature was increased to 180 °C over 2 h, then to 220 °C, and the samples were heated to dryness. Tubes were cooled to room temperature. The resulting ash was dissolved in 1 mL concentrated hydrochloric acid (HCl) and 10 mL of 5% nitric acid, mixed thoroughly, and transferred to plastic tubes for analysis. The ash solution was analyzed using ICP-MS (Agilent 7800, Agilent Technologies, Santa Clara, CA, USA) to determine the mineral content. Macrominerals were reported in ppm (mg/kg), while microminerals and trace elements were reported in ppb (µg/kg) in cheese samples.

### 2.5. Microbiological Analysis

Each 10 g sample was accurately weighed, and 90 mL of sterile physiological saline solution (0.85% NaCl) was added. The samples were homogenized using a stomacher (Interscience BagMixer 400, Saint Nom la Bretèche, France) for 2 min. Following homogenization, tenfold serial dilutions were prepared up to 10^−9^. For *Lactobacillus* enumeration, de Man Rogosa, and Sharpe Agar (MRSA, Merck, Darmstadt, Germany) was used, with incubation in an anaerobic jar at 36–37 °C for 3 days [26]. For *Lactococcus*, M17 Agar was employed, and plates were incubated at 36–37 °C under aerobic conditions for 3 days. Coliform bacteria were enumerated using Violet Red Bile Agar (VRBA, Merck, Darmstadt, Germany) and incubated at 35–37 °C for 24 h. *Staphylococcus aureus* was quantified on Baird-Parker Agar (BPA, Merck) with incubation at 37 °C for 24 h. *Escherichia coli* enumeration was performed using Chromocult Tryptone Bile X Glucuronide Agar (CTBX, Merck) at 44 °C for 24 h. Total aerobic mesophilic bacteria were determined using Plate Count Agar (PCA, Merck) at 30 °C for 48 ± 2 h. Yeast and mold were enumerated on Dichloran Rose Bengal Chloramphenicol Agar (DRBCA, Merck) at 25 °C for 3–5 days [27].

### 2.6. Lipolysis and Free Fatty Acid Analysis

The acid degree value, representing the concentration of free fatty acids, was determined using a partially modified version of the method described by Atasoy et al. [28]. Cheese samples were weighed to obtain 8–10 g of fat. The samples were placed in a stomacher bag and mixed with pure diethyl ether for 2 min using a stomacher. The extract was filtered through coarse filter paper to separate the ether-fat mixture. Pure fat was obtained by evaporating the ether using a rotary evaporator at 45 °C. A 4 g portion of the fat sample was weighed, and 40 mL of an alcohol-ether mixture (1:1) was added. Titration was performed with 0.1 N KOH prepared in alcohol, using 1% phenolphthalein as an indicator. The acid value was calculated as follows:

$$\mathrm{Acid}\;\mathrm{degree}\;\mathrm{value}\;(\mathrm{mg}\;\mathrm{KOH}\;100\;g^{-1})=\frac{V\;\left(\mathrm{mL}\right)\times N\times 56.11}{M}$$

In this formula, the following variables are defined:

V: Volume of KOH used in titration (mL); N: Actual normality of KOH used in titration; M: Mass of fat sample (g).

Cheese samples were prepared following the procedures outlined by Akalın et al. [29], and fatty acid methyl esters (FAME) were generated via methylation as described by Metcalfe and Schmitz [30]. Fatty acid composition was analyzed using gas chromatography (GC-Agilent 6890N, Agilent Technologies, Santa Clara, CA, USA) equipped with an HP-Innowax capillary column (60 m × 0.25 mm ID × 0.25 µm). The column temperature increased from 100 °C to 200 °C at a rate of 5 °C/min. Detection utilized a flame ionization detector (FID) with hydrogen and dry air at 260 °C, helium as the carrier gas (1 mL/min, 150 kPa), and an injection block temperature of 250 °C [31]. Individual fatty acids were identified by comparing their retention times with those of a FAME standard mixture. The relative composition of each fatty acid was calculated from the chromatographic peak areas and expressed as a percentage of total fatty acids.

### 2.7. Volatile Compounds Analysis

A 5.0 g sample was placed in a 40 mL headspace vial (Supelco, Bellefonte, PA, USA) and sealed with a PTFE-faced silicone septum (Supelco). The vials were placed in a thermal block (Supelco) and equilibrated at 40 °C for 30 min to facilitate the accumulation of volatile compounds in the headspace. Volatiles were extracted using a solid-phase microextraction (SPME) device (Model 57,330-U; Supelco) with CAR/PDMS fibers (75 µm, carboxen/polydimethylsiloxane), conditioned in the injection port of a gas chromatograph (6890 N; Agilent Technologies). Separation was performed on a DB-624 capillary column (30 m × 0.25 mm i.d., 1.4 µm film; Agilent J&W Scientific, Santa Clara, CA, USA) using helium as the carrier gas. Identification relied on mass spectrometry libraries (NIST, WILEY, FLAVOR) and standard reference materials [4].

### 2.8. Sensory Evaluation

Five experts from the Department of Food Engineering at Bayburt University and five Erzincan Tulum cheese producers in Erzincan, Türkiye, evaluated the experimental cheeses on the 120th day of ripening. All panelists were familiar with Tulum Cheese. Evaluation included visual characteristics, body, texture, aroma, and taste, using a 25-point scale from 5 (lowest) to 25 (highest), in accordance with the Turkish Tulum cheese guidelines. The Ethics Committee of Bayburt University granted ethical approval with decision number 374888 for the sensory analysis.

### 2.9. Statistical Analysis

Analysis of variance (ANOVA) in XLSTAT 2016–0.2, using the general linear model, assessed the effects of salt type on biochemical, physicochemical, microbiological, and sensory traits, mineral composition, free fatty acids, and volatile profiles. Bacterial counts were log_10_-transformed. The study used a randomized 4 × 2 × 2 factorial design: salt type (KS-C, SS-C, CS-C, LNaS-C), storage time (1 and 120 days), and two trials. Physicochemical and microbiological analyses, as well as fatty acid composition, were assessed on days 1 and 120 of storage. On day 120, sensory analyses, mineral composition, and aroma profile were evaluated in ready-to-eat cheeses. Four analytical replicates were performed for each measurement. One-way ANOVA and Duncan’s post hoc test (IBM SPSS Statistics 25) were used to evaluate significance at the 0.05 level.

## 3. Results and Discussion

### 3.1. Physical and Chemical Properties

Table 3 presents selected physicochemical properties of cheeses produced using different natural-source salts (Kemah spring salt, Sivas spring salt, Çankırı rock salt) and low-sodium salt.

The total dry matter content increased significantly in all cheese samples during the ripening period (*p* < 0.05), rising from an initial range of 61.36–61.65% to 68.75–70.73% at the end of ripening. This increase is attributed to the concentration effect resulting from moisture loss and the removal of the serum phase during syneresis [1], which also explains the increases observed in fat, protein, and salt contents, as well as the decrease in water activity (aw) during ripening. Comparable results have been reported previously [3,32]. At the end of ripening, significant differences were observed among the samples (*p* < 0.05); KS-C exhibited the lowest value (68.75), while SS-C and CS-C showed the highest values (70.73 and 70.44, respectively). All cheese samples met the requirements specified in the Turkish Patent and Trademark Office registration certificate and the EU registration certificate (PDO-TR-03056). Protein content also increased significantly in all samples by the end of ripening (*p* < 0.05), although no statistically significant differences were detected between samples (*p* > 0.05). The LNaS-C sample had the highest protein content at 25.185%, consistent with values reported by Arslaner and Türkmen [3] for Erzincan Tulum cheese with a similar ripening period, and higher than those reported by Hayaloglu et al. [32]. At the beginning of storage, cheese samples produced with naturally sourced salts exhibited relatively lower pH values (*p* < 0.05). The reduced total protein content in these samples is attributable to increased serum protein loss, which results from higher syneresis associated with the lower pH.

At the beginning of storage, the salt content of the cheese samples ranged from 2.39% to 3.34%, with statistically significant differences observed among samples (*p* < 0.05). This variation is likely due to incomplete diffusion of salt within the cheese matrix [33]. Natural-source salts incorporated into cheese through the dry-salting method can display differences in crystal structure and crystal size. Because the salts are not mechanically ground, they remain heterogeneous. This heterogeneity likely resulted in variable salt diffusion and differences in salt concentrations at the onset of storage. By the end of ripening, salt content increased significantly in all samples (*p* < 0.01), attributable to the moisture permeability of the goatskin packaging [3], and significant differences persisted (*p* < 0.01). SS-C and CS-C exhibited the highest salt contents (4.016% and 3.993%, respectively), while LNaS-C had the lowest (3.405%). Differences among samples are likely due to the distinct physicochemical properties of the salts used and their interactions with the cheese matrix [34].

pH values increased significantly in all cheese samples by the end of ripening, with statistically significant differences observed among samples (*p* < 0.05). Similar trends have been reported for Tulum cheeses and are associated with fungal activity [3,35,36]. Utilization of lactic acid as a carbon source by yeasts and molds, along with ammonia formation from amino acid deamination, leads to environmental alkalization and an increase in pH [3,37]. The LNaS-C sample exhibited the highest pH (5.09), while the KS-C sample had the lowest (4.86). This observation aligns with studies indicating that the use of KCl can result in higher pH values compared to NaCl [18]. However, the literature indicates that the effect of salt composition on pH is variable [11,12,18,34]. For example, in Halloumi cheese, pH increased with higher KCl content [18], whereas in Turkish white cheese with 30% KCl [11] and in Feta cheese with complete NaCl replacement [12], pH decreased. These findings demonstrate that the impact of salt composition on pH is not uniform and depends on microbial and proteolytic activity, cheese variety, production conditions, and ripening duration [34].

Water activity values decreased significantly in all cheese samples by the end of ripening (*p* < 0.05). This reduction is attributed to a decrease in the free water fraction resulting from moisture loss and increased salt concentration. While the impact of different salt types on water activity may vary due to differences in molecular weight [38], some studies have indicated that partial substitution of NaCl with KCl did not significantly affect water activity [39]. In the present study, the higher water activity observed in the LNaS-C sample was likely due to a greater free water fraction resulting from its lower salt content.

Table 2 presents the changes in total nitrogen, water-soluble nitrogen (WSN), and ripening index (RI) values of Erzincan Tulum cheese samples over a 120-day ripening period. Total nitrogen (TN) increased in all samples during the ripening period (*p* < 0.01). The WSN fraction, which includes free amino acids from casein breakdown, whey proteins, and small- to medium-molecular-weight peptides, serves as a key indicator of proteolysis in cheese [40]. WSN increased in all samples throughout ripening (*p* < 0.01), consistent with previous studies on cheeses produced from raw sheep’s milk [3,33]. Salt composition significantly affected WSN and RI values (*p* < 0.01). The KS-C control sample, with the highest sodium content, exhibited the lowest WSN and RI values (*p* < 0.01). In contrast, the LNaS-C sample, prepared with low-sodium salt and exhibiting the lowest salt and sodium content, showed higher TN, WSN and RI values on day 120 compared to cheeses produced with naturally sourced salts. These differences are attributed to variations in salt content and composition during ripening. The inverse relationship between proteolysis and salt content in cheese has been widely reported [1,41].

### 3.2. Mineral Composition and Presence of Toxic Metals

Table 4 summarizes the mineral and toxic metal analyses of Tulum cheeses produced with various types of salt.

The type of salt used significantly influenced the concentrations of minerals, except for calcium (Ca), specifically potassium (K), phosphorus (P), sodium (Na), magnesium (Mg), and boron (B), in the cheese samples (*p* < 0.01). Calcium, an essential mineral in cheese, was present at concentrations ranging from 3607.30 to 4970.50 mg/kg. No statistically significant differences in potassium content were observed among the KS-C, SS-C, and CS-C samples produced with natural-source salts. As expected, based on salt type, the highest potassium (50,105.28 mg/kg) and lowest sodium (35,464.20 mg/kg) levels were detected in the cheese sample produced with low-sodium salt (LNaS-C). The KS-C sample (PDO) exhibited higher sodium (79,274.34 mg/kg) and phosphorus (12,826.89 mg/kg) levels than the other samples (*p* < 0.01). Sodium is required for metabolic functions, including nerve transmission, muscle contraction, and maintenance of electrolyte and fluid balance [42]. The World Health Organization (WHO) recommends a maximum daily intake of 5000 mg NaCl (equivalent to 2000 mg sodium) for adults. Most individuals consume between 3600 and 6000 mg of sodium daily, with a 30% reduction targeted by 2025 [34]. Based on the sodium content determined in the dry weight of the KS-C, SS-C, CS-C, and LNaS-C cheese samples, consumption of 100 g of aged cheese would result in sodium intakes of approximately 5450.1, 4667.3, 5050.7, and 2469.4 mg, respectively.

Among the microminerals, copper (Cu), iron (Fe), and silicium (Si) were below detectable limits in all samples, while zinc (Zn) was undetectable in the SS-C and LNaS-C samples. The highest manganese (Mn) concentration (5679.75 µg/kg) was observed in the KS-C sample, and the highest zinc concentration (60,287.1 µg/kg) was found in the CS-C sample. Manganese is a critical trace mineral involved in enzyme reactions, protein metabolism, and bone formation [43]. The recommended daily intake of Mn is 2.0–3.0 mg [44]. Zinc is an essential micronutrient with multiple physiological functions and is a component of many enzymes [42]. The recommended daily intake of Zn is 12–15 mg, with a threshold of 0.3–1.0 mg/kg body weight [44]. All cheese samples represent good sources of manganese, while the KS-C and CS-C samples are also good sources of zinc. Cobalt (Co) concentrations in the cheese samples ranged from 128.20 to 161.70 µg/kg, remaining below the recommended maximum limit of 0.05 mg/kg [45]. In cheeses produced with various source salts and low-sodium salt, heavy metals such as arsenic (As), aluminum (Al), chromium (Cr), cadmium (Cd), nickel (Ni), lead (Pb), and tin (Sn) were not detected. Mercury (Hg) was present in the KS-C, SS-C, and LNaS-C samples at concentrations of 513.20 µg/kg, 1916.3 µg/kg, and 247.0 µg/kg dry weight, respectively, while it was below detectable limits in the CS-C sample. Chronic exposure to elevated mercury levels can result in severe health issues, including organ failure and cancer [46]. The FAO/WHO Expert Committee on Food Additives (JECFA) revised the provisional acceptable weekly intake for total mercury from 5 µg/kg body weight to 4 µg/kg body weight for inorganic mercury, based on updated toxicological data [47]. This corresponds to 280 µg mercury per week for an individual weighing 70 kg. According to the findings of the present study, consuming 100 g of ripened Tulum cheese would result in mercury intakes of 35.2 µg, 135.5 µg, and 17.2 µg for the KS-C, SS-C, and LNaS-C samples, respectively.

A study evaluating edible salts in Türkiye for essential and non-essential elements reported that the hazard index values for Çankırı rock salt and Sivas spring salt were below safety thresholds, and all salts were classified as low risk [48]. However, continued monitoring of heavy metal contamination in regional cheeses, including Tulum cheese, remains a significant public health concern. The observed differences in mineral composition among the cheese samples are likely due to the use of spring salts and rock salts sourced from different regions.

### 3.3. Microbiological Properties

Table 5 presents the changes in microbial load after 120 days of storage. At the conclusion of the storage period, the counts of coliforms, *E. coli*, and *Staphylococcus aureus* in the cheese samples remained below the detection limit, specifically <1, <1, and <2 log CFU/g, respectively (Table 5).

The change in the total aerobic mesophilic bacteria (TAMB) count during storage was not statistically significant. On the 120th day, the highest TAMB count was observed in the SS-C sample (8.482 log CFU/g), while the lowest was found in the LNaS-C sample (8.144 log CFU/g) (*p* < 0.05). Although the yeast and mold count decreased by the end of storage (*p* < 0.01), it remained within the range of 4.446–5.556 log CFU/g in the cheese samples on day 120 (*p* < 0.01). Yeasts play a crucial role in the development of cheese flavor and texture due to their acid-reducing, proteolytic, and lipolytic activities [49]. Studies on non-starter lactic acid bacteria (NSLAB) indicate that yeasts contribute to the development of terroir signatures in regional cheeses [50]. Free fatty acids and esters produced by yeasts are associated with fruity, cheesy, or sour flavors [50,51]. At the beginning of storage, the lowest counts of Lactococci and Lactobacilli (8.139 log CFU/g and 8.246 log CFU/g, respectively) were observed in the LNaS-C sample (*p* < 0.01). By the end of storage, the samples exhibited similar counts. Storage time had no significant effect on Lactococci and Lactobacilli counts in the LNaS-C sample. Similarly, in the KS-C control sample, no significant difference in Lactobacillus counts was detected during storage. In contrast, the SS-C and CS-C samples showed a significant decrease in Lactococci and Lactobacilli counts by day 120 (*p* < 0.05; *p* < 0.01). Regional cheese samples produced from raw milk, such as Erzincan Tulum cheese, are expected to possess a rich microflora. Consequently, cheeses made from raw milk typically exhibit a more intense and complex flavor profile compared to processed cheeses [52].

### 3.4. Lipolysis and Free Fatty Acid Composition

**Figure 2:**
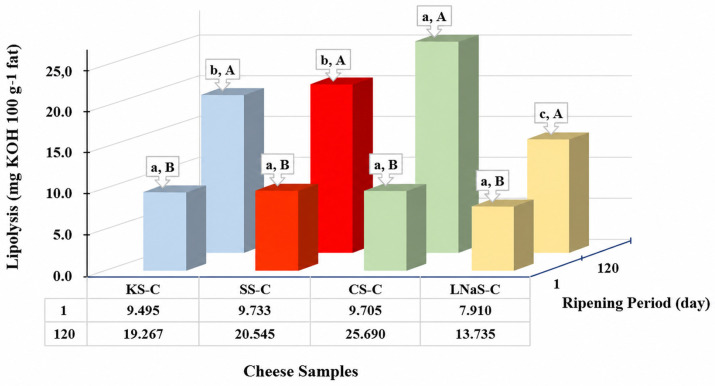
Changes in lipolysis values of Erzincan Tulum cheese samples. Different capital letters; A, B (between samples at the same storage day) and different lower letters; a–c (during storage days) in lipolysis indicate significant statistical differences (*p* < 0.05). KS-C: Kemah salt cheese; SS-C: Sivas salt cheese; CS-C: Çankırı salt cheese; LNaS-C: Low-sodium salt cheese.

Lipolysis levels were measured at multiple storage intervals in cheese samples produced with different salt types. The corresponding values for each sample are presented in Figure 2.

From the beginning to the end of ripening, lipolysis values increased significantly in all cheese samples (*p* < 0.05), indicating ongoing lipolytic activity. Initially, there were no significant differences in lipolysis among samples (*p* > 0.05), suggesting comparable initial lipolysis levels. At the end of ripening, significant differences were observed (*p* < 0.05): CS-C cheese exhibited the highest lipolysis value, LNaS-C the lowest, while KS-C and SS-C displayed similar levels and were statistically grouped together. Lipase activity in cheese originates from milk, rennet, and microbial sources and is particularly effective in generating short- and medium-chain fatty acids. These free fatty acids serve as precursors for aroma and flavor compounds [53,54]. The effect of salt composition on lipolysis varies across studies. In Feta [55] and Kefalograviera [16] cheeses, replacing NaCl with KCl in various ratios did not significantly alter lipolysis levels during ripening. Conversely, studies on Cheddar cheese reported increased lipolysis with MgCl_2_, CaCl_2_, and KCl, with MgCl_2_ and CaCl_2_ producing the most pronounced effects [56]. Additionally, some research indicates that higher salt content increases lipolysis [33]. Similarly, the LNaS-C sample, with the lowest salt concentration, showed the lowest lipolysis level in this study. In Turkish white cheese, the highest lipolysis was observed in the 50% reduced-salt sample and was also elevated in calcium-salt samples, whereas potassium- and magnesium-salt samples resulted in lower lipolysis [11]. Overall, these findings demonstrate that different salt types and concentrations can significantly influence lipase activity and lipolysis outcomes in various cheese types [33].

The fatty acid profiles of four cheese samples produced with different salt types were analyzed at the beginning and end of ripening. The analysis focused on statistically significant, technologically relevant fatty acids (Table 6). Oleic, palmitic, stearic, and myristic acids were identified as the dominant fatty acids in all samples, consistent with previous findings for Tulum cheeses [57]. Table 6 presents the changes in the fatty acid composition of Erzincan Tulum cheese samples (% of total fatty acids).

Short-chain fatty acids (SCFA, C4–C10) accounted for 4.96% to 7.17% of TFA. Capric acid (C10:0) was the predominant SCFA and contributed directly to cheese aroma [58]. At the beginning of ripening, no statistically significant differences in SCFA levels were observed among samples (*p* > 0.05). At the end of ripening, significant decreases in SCFA levels were detected in KS-C, CS-C, and LNaS-C samples (*p* < 0.05), with values declining from 6.12–7.17% to 4.96–5.53%. In contrast, SCFA levels in the SS-C sample remained stable and higher than those in other samples at the end of ripening. This reduction in SCFAs is likely attributable to their conversion into aroma compounds [59]. Although KS-C and SS-C exhibited comparable lipolysis levels at the end of ripening, SCFA levels remained stable only in the SS-C sample. This finding suggests that changes in SCFA composition cannot be explained solely by the overall extent of lipolysis. The type of salt may have contributed to the observed differences in SCFA composition by influencing the subsequent biochemical transformations of short-chain fatty acids released during lipolysis.

Medium-chain fatty acids (MCFA, C11:0-C14:1) accounted for approximately 12.49–13.45% of TFA, with no significant changes during ripening or variation among samples. Long-chain fatty acids (LCFA; ≥C15:0) comprised about 80–82% of TFA. Although MCFA and LCFA were quantitatively dominant, their contribution to cheese aroma was limited [59]. Notably, LCFA levels increased in CS-C and LNaS-C samples at the end of ripening, while no significant changes were observed in KS-C and SS-C samples (*p* < 0.05). This increase in the relative proportion of LCFA may reflect differences in the relative metabolism of fatty acid groups during ripening rather than an absolute increase in LCFA content.

Palmitic (C16:0) acid increased in CS-C and LNaS-C samples, while stearic (C18:0) acid increased in KS-C, CS-C, and LNaS-C samples at the end of ripening (*p* < 0.05). No significant changes were observed in the SS-C sample (*p* > 0.05). These findings are consistent with previously reported increases in stearic acid [60]. Oleic acid, a major LCFA, accounted for approximately 27% of TFA in all samples and remained stable during ripening (*p* > 0.05). The elevated levels of these fatty acids may result from the preferential release of these fractions by lipases after the release of short-chain fatty acids during lipolysis [53]. Similarly, the absence of significant changes in palmitic and stearic acids in the SS-C sample, together with the stable SCFA levels observed during ripening, suggests that the release and subsequent transformation of fatty acids may have differed from those in the other salt treatments. Polyunsaturated fatty acids (PUFA) comprised about 5% of TFA, with linoleic (C18:2n6c), α-linolenic (C18:3n3), and linolelaidic (C18:2n6t) acids as the main components. At the end of ripening, PUFA content decreased only in the KS-C sample (*p* < 0.05), with no significant changes in other samples. Additionally, saturated fatty acids (SFA), monounsaturated fatty acids (MUFA), and medium-chain fatty acids (MCFA) did not change significantly at the end of ripening (*p* > 0.05). Overall, variations in fatty acid composition among cheese samples are likely attributable to microbial and enzymatic processes influenced by the type of salt used [53,58].

### 3.5. Volatile Compound Profile of Erzincan Tulum Cheese Samples

Salt concentration significantly affects cheese composition, enzymatic activities, and biochemical modifications [41]. While reduced salt content is known to enhance proteolysis by increasing microbial and enzymatic activity, research on its effect on lipolysis in semi-hard cheeses remains limited and inconclusive [4]. Cheese flavor, developed through biochemical reactions such as lipolysis and proteolysis, is a primary determinant of consumer preference and acceptance [61]. SPME-GC-MS analysis of Tulum cheese samples identified 50 volatile compounds, comprising 8 aldehydes and ketones, 10 alcohols, 14 esters, 9 acids, 3 terpenes, and 6 additional compounds (Table 7).

Significant differences in carbonyl compound content were observed among cheeses produced with various source salts and low-sodium salt (*p* < 0.05; *p* < 0.01). Compared to samples produced with natural-source salts, concentrations of 2-propanone, 2-butanone, 2,3-butanedione, and acetoin were higher in LNaS-C cheese (*p* < 0.01). Ketones, formed through the oxidation and decarboxylation of free fatty acids, strongly influence cheese aroma even at low concentrations due to their pungent odors and low sensory thresholds, contributing to buttery and cooked notes [4]. LNaS-C, which contained the highest total carbonyl compounds, was among the two samples with the highest taste and odor scores in the sensory panel. The concentration of 2-methyl-3-heptanone was highest in the KS-C control sample (*p* < 0.01). 2-methylbutanal was the only aldehyde detected in the KS-C, CS-C, and LNaS-C samples, with the highest concentration in KS-C. The low concentration of carbonyl compounds after storage is attributed to the rapid metabolism of ketones to alcohols, the dehydrogenation of aldehydes into acids, and the subsequent formation of esters from alcohols and carboxylic acids through alcoholysis and esterification reactions [4,5]. This mechanism may also explain the lower total carbonyl compound content (~4.173%) and the higher total ester content (~20.11%) observed in the SS-C sample, suggesting that the conversion of carbonyl compounds into subsequent volatile compounds may have occurred to a greater extent during ripening in this treatment.

Alcoholic volatiles constituted the most abundant group within the volatile fraction of cheeses, followed by carboxylic acids. This distribution is characteristic of cheeses produced from raw milk, which are known for their high microbial diversity [3,62]. Alcohols are formed through lactose and amino acid metabolism, dehydrogenation of aldehydes and ketones, and degradation of linoleic and linolenic acids in the cheese matrix [63]. Among the ten alcohols identified, ethyl alcohol, 2-butanol, 2-methylpropanol, 3-methylbutanol, and 2-phenylethanol were most prominent across all cheese samples (Table 7). Ethyl alcohol and 3-methylbutanol were present at the highest concentrations. The type of salt used significantly affected the concentration of alcoholic volatiles (*p* < 0.05, *p* < 0.01). Aged Tulum cheeses produced with low-sodium salt (LNaS-C) exhibited the highest levels of 2-butanol, 2-methylpropanol, 3-methylbutanol, and 2-phenylethanol (*p* < 0.05, *p* < 0.01). The concentrations of 2-butanol, 2-methylpropanol, 3-methylbutanol, and benzyl alcohol were similar in aged cheeses produced with natural-source salts (KS-C, SS-C, CS-C). Within cheeses produced with natural-source salts, the KS-C control sample had the lowest ethyl alcohol and total volatile alcohol concentrations (*p* < 0.01), likely due to the lowest yeast and mold count observed in KS-C at the beginning of storage. The biosynthesis of primary and aromatic alcohols, as well as related carboxylic acids, is primarily dependent on mold and yeast metabolism [5]. The presence of alcohols such as 2-methylpropanol, 3-methylbutanol, and 2-phenylethanol, together with carboxylic acids such as 2-methylpropanoic acid, suggests the reduction in branched-chain and aromatic free amino acids [5]. The highest proportions of these secondary alcohols and 2-methylpropanoic acid were detected in the LNaS-C sample, which also exhibited higher WSN and RI values than other cheese samples (*p* < 0.01; *p* < 0.05). This finding suggests that the enhanced proteolysis observed in the LNaS-C sample may have increased the availability of free amino acids, which could subsequently be converted into alcohols by microbial metabolism during ripening. Although alcohols have high detection thresholds and therefore have a limited direct impact on cheese flavor, they contribute to the distinctive, recognizable aroma of traditional cheeses [3,62]. Comparison with other Tulum cheeses produced from raw sheep’s milk revealed differences in the concentrations and dominant types of alcoholic volatile compounds [64].

Esters that impart sweet and fruity notes to cheese are synthesized via esterification or alcoholysis reactions [3,5]. The salt variable significantly influenced the ester profile of all esters except ethyl isobutyrate in the cheese samples (*p* < 0.05; *p* < 0.01). In the present study, the dominant esters identified in Erzincan Tulum cheese included ethyl acetate, ethyl butyrate, 3-methyl butyl acetate, and hexanoic acid ethyl ester (Table 7). The lowest concentrations of the latter three compounds were detected in the low-sodium LNaS-C sample. The SS-C sample exhibited the highest concentrations of ethyl acetate and ethyl butyrate (10.76% and 5.61%, respectively), as well as the highest total ester concentration. Propanoic acid ethyl ester was detected exclusively in the SS-C sample. These compounds contribute to the flavor balance of cheese by moderating the acidity of carboxylic acids [62] but are reported to cause sensory defects when present in excess [58]. The KS-C control sample contained the highest concentrations of 3-methyl butyl acetate, 2-phenylethyl acetate, and methyl hexadecanoate (*p* < 0.01 and *p* < 0.05, respectively). No significant difference was observed between cheeses produced with natural-source salts in hexanoic acid ethyl ester and ethyl caprylate concentrations.

Among the nine carboxylic acids identified in the cheese samples, acetic acid, 2-methylpropanoic acid, butanoic acid, pentanoic acid, hexanoic (caproic) acid, and octanoic (caprylic) acid were the most prevalent (Table 7). The application of different natural-source salts significantly influenced the carboxylic acid profile, contributing to the cheese’s sour, fatty, fruity, and sharp sensory attributes (*p* < 0.05; *p* < 0.01). No significant differences in acetic acid and pentanoic acid concentrations were observed among aged cheeses produced with natural-source salts (KS-C, SS-C, CS-C). However, the KS-C control sample exhibited the highest concentrations of propanoic acid, butanoic acid, caproic acid, and caprylic acid, distinguishing it from other samples produced with natural-source salts (*p* < 0.01). The LNaS-C sample contained the lowest levels of acetic acid, butanoic acid, caproic acid, and caprylic acid. In contrast, LNaS-C, produced with low-sodium salt, showed the highest abundance of 2-methylpropanoic acid and pentanoic acid (*p* < 0.01). Short-chain fatty acids with low detection thresholds are regarded as the primary contributors to the distinctive flavor of traditional sheep’s milk cheeses [36].

Within the terpene group, *α*-pinene, *β*-pinene, and *d*-Limonene were identified as the primary monoterpenes present in Tulum cheese samples (Table 7). No significant differences in monoterpene concentrations were observed among the samples (*p* > 0.05). The practice of feeding livestock in highland areas with green fodder is a major source of volatile compounds, including terpenes, that contribute to cheese flavor [3].

Variations in the volatile component composition among the samples are attributed to differences in the mineral compositions of the salts used during production. Analysis of the macro- and micro-mineral content in cheese samples produced with natural-source salts (Table 4) shows that the KS-C control sample contains higher levels of phosphorus, sodium, and manganese. Previous research has demonstrated that the specific volatile component profile can vary according to factors such as milk source, temperature, and ripening conditions [4]. Furthermore, prior studies have shown that differences in coagulants significantly influence aroma composition [3]. The present findings suggest that differences in the mineral composition of the salts may have modulated biochemical reactions, including proteolysis and lipolysis, during ripening, thereby contributing to the differences observed in the aroma profiles of the cheeses. The origin and composition of salt, the type of milk, the technological processes applied to the milk, the coagulant used, the packaging material, and ripening conditions collectively determine the characteristic qualities of all cheeses after ripening, particularly those with protected designation of origin (PDO).

### 3.6. Sensory Evaluation of Erzincan Tulum Cheeses

**Figure 3:**
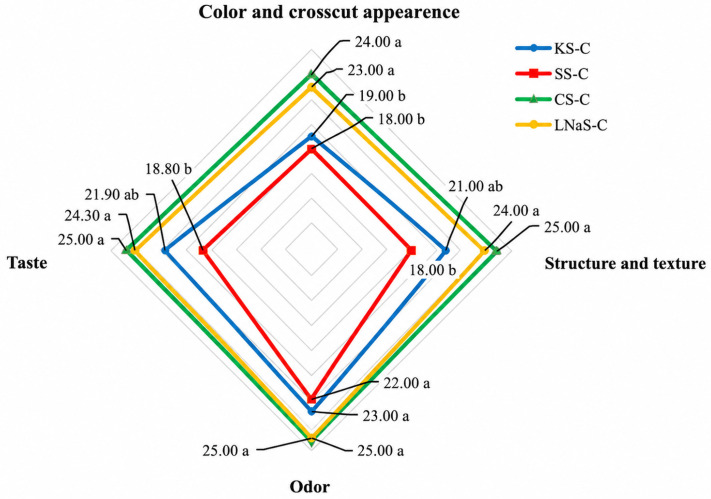
Sensory properties in Erzincan Tulum cheese samples. Different lower letters given on the chart indicate significant differences (*p* < 0.05). KS-C: Kemah salt cheese; SS-C: Sivas salt cheese; CS-C: Çankırı salt cheese; LNaS-C: Low-sodium salt cheese.

Panelists evaluated the sensory properties of Erzincan Tulum cheese samples using a 100-point scale, with 25 points assigned to each parameter: color and cross-sectional appearance, structure and texture, taste, and odor (Figure 3). Statistically significant differences were observed among the samples for all parameters except odor (*p* < 0.05). The CS-C sample achieved the highest total sensory score (99.0), followed by LNaS-C (96.30) and KS-C (84.90), while the SS-C sample received the lowest score (76.80) (Figure 3). For taste, the CS-C sample obtained the highest score (25.00), statistically similar to LNaS-C (24.30) and KS-C (21.90) (*p* > 0.05). The SS-C sample had the lowest taste score (18.80), which was significantly lower than those of the other samples (*p* < 0.05). The elevated sensory acceptance values for CS-C and LNaS-C are likely due to a more balanced distribution of ester, ketone, and alcohol compounds, which contribute to desirable aroma formation [5]. Volatile organic compound analysis revealed high concentrations of ethyl acetate and ethyl butyrate in the SS-C sample, distinguishing it from the other samples. Ethyl acetate levels in the SS-C sample were similar to those reported for KS-C, CS-C, and LNaS-C by Gezginc et al. [64], but a markedly higher accumulation was observed in SS-C. The literature indicates that esters, such as ethyl acetate, contribute to a fruity aroma, although excessive accumulation may result in sensory defects [58]. Likewise, reduced overall acceptability in Tulum cheeses ripened in plastic barrels has been linked to increased ester accumulation [65]. Compared with values reported by Tekin and Guler [65], the present study identified a higher acetic acid concentration in the SS-C sample. Acetic acid is associated with sharp, sour, and vinegary sensory perceptions. Additionally, substituting NaCl with MgCl_2_ has been reported to negatively impact sensory acceptance by increasing sour, bitter, metallic, and soapy taste perceptions in cheeses [66]. Collectively, these findings suggest that the elevated ester accumulation, along with higher acetic acid and magnesium levels in the SS-C sample, may have adversely affected sensory acceptance by producing a sharp, sour, and unbalanced taste profile.

Regarding color and cross-sectional appearance, the CS-C sample received the highest score (24.00), while the SS-C sample received the lowest (18.00). Statistically, the CS-C and LNaS-C samples were similar, whereas KS-C and SS-C samples had lower scores. The high dry matter and salt content, along with low water activity in the SS-C sample, may have contributed to a more compact structure. The literature indicates that monovalent salts are associated with greater light reflection and increased whiteness than divalent salts [12]. Conversely, the elevated magnesium content in the SS-C sample may have reduced light reflection, resulting in a darker appearance.

For structure and texture, the CS-C sample achieved the highest score (25.00), while the SS-C sample had the lowest (18.00). These differences are likely attributable to the type of salt used and the resulting mineral distribution. Elevated salt, pH, and magnesium levels, particularly in the SS-C sample, may have contributed to a more brittle, fragile texture by altering the cheese matrix. The literature indicates that high salt content can influence casein hydration, protein network structure, and elasticity, thereby increasing brittleness [67]. Additionally, brittleness is reported to increase and texture scores to decrease in cheeses containing MgCl_2_ [56]. Collectively, these findings suggest that the low structure and texture score in the SS-C sample may result from the combined effects of high salt, pH, and magnesium levels.

## 4. Conclusions

The authenticity of Protected Designation of Origin (PDO) cheeses is defined by their region of origin, traditional production methods, and human factors affecting each stage of the production process. Salt content and type are key components of the PDO Erzincan Tulum cheese production method. Prior to this study, the effects of different natural salts and low-sodium salt formulations on the quality characteristics of this cheese had not been systematically investigated. The findings show that the use of various natural salts, such as natural-source salt and rock salt, or low-sodium salt, significantly affects the chemical composition, lipolysis levels, selected ripening criteria (WSN, RI), aroma, mineral composition, and ultimately the sensory characteristics of PDO Erzincan Tulum cheese, even though these salt formulations are technologically suitable for Tulum cheese production. Cheeses produced with a low-sodium salt formulation had the lowest sodium and highest potassium content, the highest proteolysis level, and the highest total carbonyl compound content. In contrast, the CS application demonstrated the highest lipolytic activity and achieved the highest overall sensory acceptance, while the SS application was characterized by the highest total ester content, which correlated with lower sensory acceptance. The use of low-sodium salt resulted in a sodium content of approximately 2469.4 mg/100 g, demonstrating that low-sodium Tulum cheese can be produced without compromising product quality. From an industrial perspective, CS may be a viable option for producers aiming to maximize consumer acceptance, while the use of low-sodium salt offers a practical strategy for producing healthier Tulum cheese for consumers requiring sodium-restricted diets. Since only a single low-sodium blend of natural rock salt and KCl (50:50, LNaS) was evaluated in the present study, future studies should investigate the effects of different blending ratios and alternative mineral salt formulations such as MgCl_2_, KCl, and CaCl_2_ on the quality characteristics of Tulum cheese.

## Figures and Tables

**Table 1:** Some physicochemical and microbiological properties of raw milk and salt samples.

Properties	Milk	Salt Samples *			
		KS	SS	CS	LNaS
Nonfat dry matter (%)	11.50	-	-	-	-
Fat (%)	8.79	-	-	-	-
Protein (%)	4.39	-	-	-	-
Acidity (%LA)	0.20	-	-	-	-
pH	6.60	-	-	-	-
Freezing point	−0.620	-	-	-	-
Density (g/mL)	1.0376	-	-	-	-
Total aerobic mesophilic bacteria	8 × 10^4^	1.97 × 10^4^	1.66 × 10^4^	1.55 × 10^3^	1.33 × 10^3^
Yeast and Molds	-	<10^2^	<10^2^	<10^2^	<10^2^
Coliforms	-	<10	<10	<10	<10
*E. coli*	-	<10	<10	<10	<10
*Staphylococcus aureus*	-	<10^2^	<10^2^	<10^2^	<10^2^

* KS: Kemah natural spring salt; SS: Sivas natural spring salt; CS: Çankırı natural rock salt; LNaS: Low-sodium natural salt (50% natural rock salt; 50% KCl).

**Table 2:** Sample labels, sample descriptions, and salt treatments used in the study.

Sample Label	Sample Description	Salt Treatments
KS-C	Erzincan Tulum Cheese (control)	%2 Kemah natural spring salt
SS-C	Sivas Spring Salt Cheese	%2 Sivas natural spring salt
CS-C	Çankırı Natural Rock Salt Cheese	%2 Çankırı natural rock salt
LNaS-C	Low-Sodium Natural Salt Cheese	1% Natural rock salt + 1% KCl (50:50, *w*/*w*)

**Table 3:** Changes in some physical and chemical properties of Erzincan Tulum cheese samples *.

Properties	Days	Cheese Samples				*p*-Value
		KS-C	SS-C	CS-C	LNaS-C	
Total solids	1	61.645 ± 0.087 ^a, B^	61.360 ± 0.092 ^a, B^	61.530 ± 0.427 ^a, B^	61.460 ± 0.046 ^a, B^	ns
	120	68.750 ± 0.277 ^c, A^	70.735 ± 0.087 ^a, A^	70.445 ± 0.110 ^a, A^	69.630 ± 0.196 ^b, A^	**
	*p*-value	**	**	**	**	
Fat	1	36.00 ± 0.000 ^a, B^	36.25 ± 0.289 ^a, B^	36.25 ± 0.200 ^a, B^	36.00 ± 0.000 ^a, B^	ns
	120	40.00 ± 0.577 ^c, A^	42.25 ± 0.200 ^b, A^	43.00 ± 0.480 ^a, A^	40.37 ± 0.478 ^c, A^	**
	*p*-value	**	**	**	**	
Protein	1	22.828 ± 0.656 ^ab, B^	22.333 ± 0.527 ^ab, B^	21.845 ± 0.059 ^b, B^	23.261 ± 0.604 ^a, B^	*
	120	24.368 ± 0.615 ^a, A^	24.196 ± 0.387 ^a, A^	24.049 ± 1.064 ^a, A^	25.185 ± 0.173 ^a, A^	ns
	*p*-value	*	**	**	**	
Salt	1	3.338 ± 0.009 ^a, B^	2.386 ± 0.005 ^d, B^	2.449 ± 0.036 ^c, B^	2.509 ± 0.011 ^b, B^	**
	120	3.917 ± 0.023 ^b, A^	4.016 ± 0.014 ^a, A^	3.993 ± 0.006 ^a, A^	3.405 ± 0.000 ^c, A^	**
	*p*-value	**	**	**	**	
pH	1	4.460 ± 0.034 ^a, B^	4.680 ± 0.012 ^a, B^	4.677 ± 0.029 ^a, B^	4.705 ± 0.017 ^a, B^	ns
	120	4.860 ± 0.00 ^c, A^	5.020 ± 0.046 ^b, A^	4.998 ± 0.060 ^b, A^	5.090 ± 0.000 ^a, A^	**
	*p*-value	**	**	**	**	
aw	1	0.889 ± 0.0023 ^ab, A^	0.886 ± 0.0005 ^b, A^	0.885 ± 0.0017 ^b, A^	0.892 ± 0.0017 ^a, A^	**
	120	0.866 ± 0.0014 ^b, B^	0.854 ± 0.0017 ^d, B^	0.859 ± 0.0014 ^c, B^	0.877 ± 0.0022 ^a, B^	**
	*p*-value	**	**	**	**	
T N	1	3.578 ± 0.102 ^ab, B^	3.501 ± 0.083 ^bc, B^	3.424 ± 0.009 ^c, B^	3.646 ± 0.095 ^a, B^	*
	120	3.819 ± 0.096 ^ab, A^	3.793 ± 0.061 ^ab, A^	3.770 ± 0.167 ^b, A^	3.948 ± 0.027 ^a, A^	*
	*p*-value	**	**	**	**	
WSN	1	0.263 ± 0.012 ^c, B^	0.326 ± 0.007 ^a, B^	0.246 ± 0.003 ^d, B^	0,290 ± 0.005 ^b, B^	**
	120	0.370 ± 0.007 ^c, A^	0.431 ± 0.049 ^ab, A^	0.386 ± 0.041 ^bc, A^	0.471 ± 0.023 ^a, A^	**
	*p*-value	**	**	**	**	
RI	1	7.379 ± 0.536 ^c, B^	9.313 ± 0.422 ^a, B^	7.171 ± 0.085 ^c, B^	7.949 ± 0.314 ^b, B^	**
	120	9.705 ± 0.402 ^c, A^	11.359 ± 1.348 ^ab, A^	10.215 ± 0.643 ^bc, A^	11.935 ± 0.513 ^a, A^	**
	*p*-value	**	**	**	**	

Different lower letters (between samples at the same storage day) in the same line and different capital letters (during storage days) in the same column indicate significant differences (* *p* < 0.05; ** *p* < 0.01; ns: not significant). KS-C: Kemah salt cheese; SS-C: Sivas salt cheese; CS-C: Çankırı salt cheese; LNaS-C: Low-sodium salt cheese. TN: Total nitrogen, WSN: Water Soluble Nitrogen, RI: Ripening Index.

**Table 4:** Mineral and heavy metal content of Erzincan Tulum cheese samples.

**Macro Minerals** **(mg/kg Dry Weight)**	**Cheese Samples**				
	**KS-C**	**SS-C**	**CS-C**	**LNaS-C**	***p*-Value**
Ca	4428.23 ± 179.5 ^a^	4970.50 ± 2184.4 ^a^	4236.48 ± 131.7 ^a^	3607.30 ± 242.4 ^a^	ns
K	2331.89 ± 106.4 ^b^	2273.05 ± 77.8 ^b^	2028.17 ± 182.8 ^b^	50,105.28 ± 590.6 ^a^	**
P	12,826.89 ± 282.7 ^a^	12,067.38 ± 1231.2 ^ab^	8927.28 ± 832.0 ^c^	11,162.35 ± 789.0 ^b^	**
Na	79,274.34 ± 360.2 ^a^	65,983.36 ± 507.4 ^c^	71,697.12 ± 788.6 ^b^	35,464.20 ± 954.0 ^d^	**
Mg	1159.81 ± 118.3 ^ab^	1265.31 ± 525.6 ^a^	805.65 ± 45.71 ^b^	989.55 ± 98.7 ^ab^	**
B	14,974.76 ± 523.1 ^a^	10,555.64 ± 335.5 ^b^	15,978.84 ± 239.2 ^a^	13,082.71 ± 166.7 ^ab^	*
**Micro Minerals** **(µg/kg Dry Weight)**	**KS-C**	**SS-C**	**CS-C**	**LNaS-C**	***p*-Value**
Cu	nd	nd	nd	nd	-
Fe	nd	nd	nd	nd	-
Mn	5679.75 ± 111.1 ^a^	3604.30 ± 1236.22 ^c^	4437.75 ± 124.30 ^bc^	4740.70 ± 347.10 ^ab^	**
Si	nd	nd	nd	nd	-
Zn	17,174.5 ± 7015.0 ^b^	nd ^c^	60,287.1 ± 1445.5 ^a^	nd ^c^	**
Co	140.25 ± 72.80 ^a^	161.70 ± 64.32 ^a^	128.20 ± 19.05 ^a^	118.00 ± 40.07 ^a^	ns
**Heavy Metals** **(µg/kg Dry Weight)**	**KS-C**	**SS-C**	**CS-C**	**LNaS-C**	***p*** **-Value**
As	nd	nd	nd	nd	-
Al	nd	nd	nd	nd	-
Cr	nd	nd	nd	nd	-
Cd	nd	nd	nd	nd	-
Hg	513.20 ± 0.78 ^b^	1916.3 ± 0.529 ^a^	nd ^d^	247.00 ± 0.028 ^c^	**
Ni	nd	nd	nd	nd	-
Pb	nd	nd	nd	nd	-
Sn	nd	nd	nd	nd	-

Mean values followed by different letters in the same column indicate significant differences (** *p* < 0.01; * *p* < 0.05; ns: no significance; nd: not detected. KS-C: Kemah salt cheese; SS-C: Sivas salt cheese; CS-C: Çankırı salt cheese; LNaS-C: Low-sodium salt cheese.

**Table 5:** Changes in microbiological properties of Erzincan Tulum cheese (log CFU/g).

	Cheese Samples					
	Days	KS-C	SS-C	CS-C	LNaS-C	*p*-Value
TAMB	1	8.163 ± 0.067 ^a, A^	8.337 ± 0.088 ^a, A^	8.346 ± 0.030 ^a, A^	8.334 ± 0.312 ^a, A^	ns
	120	8.272 ± 0.076 ^ab, A^	8.482 ± 0.117 ^a, A^	8.236 ± 0.094 ^ab, A^	8.144 ± 0.259 ^b, A^	*
	*p*-value	ns	ns	ns	ns	
Coliforms	1	3.222 ± 0.292 ^a, A^	2.956 ± 0.208 ^ab, A^	2.756 ± 0.208 ^bc, A^	2.570 ± 0.198 ^c, A^	**
	120	<1 ^B^	<1 ^B^	<1 ^B^	<1 ^B^	-
	*p*-value	**	**	**	**	
*E. coli*	1	1.189 ± 0.219 ^a, A^	2.095 ± 0.457 ^a, A^	<1 ^b^	<1 ^b^	**
	120	<1 ^B^	<1 ^B^	<1	<1	-
	*p*-value	**	**	-	-	
*S. aureus*	1	2.602 ± 0.215 ^b, A^	3.083 ± 0.426 ^a, A^	2.952 ± 0.039 ^a, A^	2.588 ± 0.128 ^b, A^	*
	120	<2 ^B^	<2 ^B^	<2 ^B^	<2 ^B^	-
	*p*-value	**	**	**	**	
Yeast-mold	1	5.844 ± 0.036 ^bc, A^	6.014 ± 0.141 ^ab, A^	6.045 ± 0.027 ^a, A^	5.722 ± 0.177 ^c, A^	**
	120	4.966 ± 0.091 ^b, B^	4.672 ± 0.170 ^c, B^	4.446 ± 0.036 ^c, B^	5.556 ± 0.229 ^a, B^	**
	*p*-value	**	**	**	ns	
*Lactococcus*	1	8.283 ± 0.073 ^ab, A^	8.403 ± 0.051 ^a, A^	8.414 ± 0.173 ^a, A^	8.139 ± 0.092 ^b, A^	**
	120	7.528 ± 0.202 ^a, B^	7.732 ± 0.289 ^a, B^	7.818 ± 0.149 ^a, B^	7.880 ± 0.252 ^a, A^	ns
	*p*-value	**	*	**	ns	
*Lactobacillus*	1	8.298 ± 0.046 ^ab, A^	8.486 ± 0.096 ^a, A^	8.485 ± 0.157 ^a, A^	8.246 ± 0.182 ^b, A^	*
	120	8.029 ± 0.206 ^a, A^	8.071 ± 0.100 ^a, B^	8.039 ± 0.175 ^a, B^	8.119 ± 0.094 ^a, A^	ns
	*p*-value	ns	**	**	ns	

Different lower letters (between samples on the same storage day) in the same line and different capital letters (during storage days) in the same column indicate significant differences (* *p* < 0.05; ** *p* < 0.01; ns: not significant). KS-C: Kemah salt cheese; SS-C: Sivas salt cheese; CS-C: Çankırı salt cheese; LNaS-C: Low-sodium salt cheese.

**Table 6:** Changes in the fatty acid composition of Erzincan Tulum cheese (% of total fatty acids).

		Cheese Samples				
Fatty Acid	Days	KS-C	SS-C	CS-C	LNaS-C	*p*-Value
C4:0	1	0.808 ± 0.148 ^b, A^	1.049 ± 0.031 ^a, A^	1.080 ± 0.148 ^a, A^	0.996 ± 0.023 ^ab, A^	*
	120	0.521 ± 0.004 ^a, B^	0.734 ± 0.405 ^a, A^	0.581 ± 0.065 ^a, B^	0.535 ± 0.139 ^a, B^	ns
	*p*-value	**	ns	**	**	
C6:0	1	0.878 ± 0.172 ^b, A^	1.130 ± 0.057 ^a, A^	1.187 ± 0.029 ^a, A^	1.087 ± 0.084 ^a, A^	*
	120	0.587 ± 0.115 ^b, B^	1.044 ± 0.037 ^a, A^	0.704 ± 0.141 ^b, B^	0.785 ± 0.080 ^b, B^	**
	*p*-value	*	ns	**	**	
C8:0	1	0.989 ± 0.128 ^b, A^	1.136 ± 0.100 ^ab, A^	1.180 ± 0.024 ^a, A^	1.147 ± 0.097 ^ab, A^	*
	120	0.779 ± 0.078 ^c, B^	1.146 ± 0.062 ^a, A^	0.904 ± 0.040 ^bc, B^	0.932 ± 0.088 ^b, B^	**
	*p*-value	*	ns	**	*	
C10:0	1	3.451 ± 0.200 ^a, A^	3.716 ± 0.089 ^a, A^	3.721 ± 0.049 ^a, A^	3.706 ± 0.222 ^a, A^	ns
	120	3.075 ± 0.115 ^b, B^	3.714 ± 0.115 ^a, A^	3.216 ± 0.192 ^b, B^	3.281 ± 0.155 ^b, B^	**
	*p*-value	*	ns	*	*	
C12:0	1	2.493 ± 0.046 ^a, A^	2.548 ± 0.023 ^a, A^	2.557 ± 0.023 ^a, A^	2.581 ± 0.110 ^a, A^	ns
	120	2.362 ± 0.045 ^b, B^	2.562 ± 0.077 ^a, A^	2.343 ± 0.131 ^b, A^	2.399 ± 0.068 ^ab, A^	*
	*p*-value	*	ns	ns	ns	
C14:0	1	10.395 ± 0.044 ^a, A^	10.375 ± 0.032 ^a, A^	10.375 ± 0.107 ^a, A^	10.517 ± 0.278 ^a, A^	ns
	120	10.432 ± 0.365 ^a, A^	10.365 ± 0.211 ^a, A^	9.951 ± 0.441 ^a, A^	10.247 ± 0.208 ^a, A^	ns
	*p*-value	ns	ns	ns	ns	
C16:0	1	27.287 ± 0.388 ^a, A^	27.012 ± 0.098 ^a, A^	26.930 ± 0.252 ^a, B^	26.877 ± 0.139 ^a, B^	ns
	120	27.585 ± 0.109 ^a, A^	26.737 ± 0.355 ^b, A^	27.971 ± 0.369 ^a, A^	27.744 ± 0.372 ^a, A^	**
	*p*-value	ns	ns	*	*	
C18:0	1	16.054 ± 0.270 ^a, B^	15.780 ± 0.054 ^ab, A^	15.684 ± 0.063 ^ab, B^	15.155 ± 0.777 ^b, B^	*
	120	16.664 ± 0.259 ^a, A^	16.135 ± 0.252 ^b, A^	16.347 ± 0.148 ^ab, A^	16.409 ± 0.338 ^ab, A^	*
	*p*-value	*	ns	*	*	
C18:1n9c	1	27.560 ± 0.191 ^ab, A^	27.353 ± 0.075 ^b, A^	27.462 ± 0.259 ^ab, A^	27.774 ± 0.011 ^a, A^	*
	120	27.841 ± 0.436 ^a, A^	27.542 ± 0.278 ^a, A^	27.043 ± 0.789 ^a, A^	27.531 ± 0.494 ^a, A^	ns
	*p*-value	ns	ns	ns	ns	
C18:2n6t	1	1.074 ± 0.050 ^a, A^	1.062 ± 0.059 ^a, A^	0.966 ± 0.040 ^a, A^	1.031 ± 0.082 ^a, A^	ns
	120	1.041 ± 0.070 ^a, A^	1.038 ± 0.057 ^a, A^	1.027 ± 0.066 ^a, A^	1.047 ± 0.030 ^a, A^	ns
	*p*-value	ns	ns	ns	ns	
C18:2n6c	1	2.161 ± 0.033 ^a, A^	2.132 ± 0.004 ^a, A^	2.149 ± 0.014 ^a, A^	2.187 ± 0.062 ^a, A^	ns
	120	2.099 ± 0.032 ^b, A^	2.180 ± 0.046 ^a, A^	2.143 ± 0.042 ^ab, A^	2.174 ± 0.039 ^ab, A^	*
	*p*-value	ns	ns	ns	ns	
C18:3n3	1	1.562 ± 0.013 ^ab, A^	1.526 ± 0.020 ^b, A^	1.554 ± 0.011 ^ab, A^	1.590 ± 0.033 ^a, A^	*
	120	1.501 ± 0.019 ^a, B^	1.548 ± 0.018 ^a, A^	1.495 ± 0.086 ^a, A^	1.549 ± 0.039 ^a, A^	ns
	*p*-value	**	ns	ns	ns	
SFA	1	66.038 ± 0.120 ^a, A^	66.370 ± 0.164 ^a, A^	66.296 ± 0.233 ^a, A^	65.675 ± 0.176 ^b, A^	**
	120	65.738 ± 0.313 ^a, A^	66.011 ± 0.241 ^a, A^	65.706 ± 0.701 ^a, A^	66.031 ± 0.325 ^a, A^	ns
	*p*-value	ns	ns	ns	ns	
MUFA	1	28.670 ± 0.165 ^b, A^	28.403 ± 0.079 ^b, A^	28.557 ± 0.235 ^b, A^	29.032 ± 0.187 ^a, A^	*
	120	28.910 ± 0.459 ^a, A^	28.731 ± 0.253 ^a, A^	28.118 ± 0.886 ^a, A^	28.698 ± 0.418 ^a, A^	ns
	*p*-value	ns	ns	ns	ns	
PUFA	1	5.292 ± 0.092 ^a, A^	5.194 ± 0.069 ^a, A^	5.147 ± 0.027 ^a, A^	5.292 ± 0.162 ^a, A^	ns
	120	5.153 ± 0.037 ^a, B^	5.245 ± 0.106 ^a, A^	5.176 ± 0.182 ^a, A^	5.272 ± 0.093 ^a, A^	ns
	*p*-value	*	ns	ns	ns	
SCFA	1	6.125 ± 0.643 ^a, A^	7.031 ± 0.271 ^a, A^	7.167 ± 0.091 ^a, A^	6.936 ± 0.424 ^a, A^	ns
	120	4.961 ± 0.297 ^b, B^	6.638 ± 0.249 ^a, A^	5.405 ± 0.429 ^b, B^	5.533 ± 0.222 ^b, B^	**
	*p*-value	*	ns	**	**	
MCFA	1	13.103 ± 0.050 ^a, A^	13.141 ± 0.039 ^a, A^	13.147 ± 0.131 ^a, A^	13.449 ± 0.330 ^a, A^	ns
	120	12.987 ± 0.314 ^a, A^	13.145 ± 0.294 ^a, A^	12.498 ± 0.584 ^a, A^	12.855 ± 0.268 ^a, A^	ns
	*p*-value	ns	ns	ns	ns	
LCFA	1	80.772 ± 0.670 ^a, A^	79.795 ± 0.247 ^a, A^	79.686 ± 0.075 ^a, B^	79.615 ± 0.742 ^a, B^	ns
	120	81.852 ± 0.598 ^a, A^	80.205 ± 0.139 ^b, A^	81.098 ± 0.727 ^ab, A^	81.612 ± 0.406 ^a, A^	*
	*p*-value	ns	ns	*	**	

Different lower letters (between samples at the same storage day) in the same line and different capital letters (during storage days) in the same column indicate significant differences (* *p* < 0.05; ** *p* < 0.01; ns: not significant). SCFA: short-chain fatty acids (C4:0–C10:0); MCFA: medium-chain fatty acids (C11:0–C14:1); LCFA: long-chain fatty acids (≥C15:0). SFA: saturated fatty acids; MUFA: monounsaturated fatty acids; PUFA: polyunsaturated fatty acids. KS-C: Kemah salt cheese; SS-C: Sivas salt cheese; CS-C: Çankırı salt cheese; LNaS-C: Low-sodium salt cheese.

**Table 7:** Volatile profile of Erzincan Tulum cheese samples at the end of storage.

RT	Volatile Compounds	Cheese Samples					
		KS-C	SS-C	CS-C	LNaS-C		Odor and Flavor Description ***
	Aldehydes and Ketones					*p*-value	
0.02	2-propanone	1.290 ± 0.036 b	1.193 ± 0.055 c	1.270 ± 0.020 b	1.493 ± 0.0208 a	**	Pungent
0.60	2-butanone	0.723 ± 0.112 c	nd d	1.040 ±0.160 b	2.036 ± 0.136 a	**	Fragrant, Fruit, Pleasant
0.80	2-methyl butanal	0.240 ± 0.000 a	nd c	0.150 ± 0.069 b	0.203 ± 0.040 b	**	Almond, Cocoa, Fermented, Hazelnut
1.60	2-Pentanone	0.140 ± 0.002 a	nd b	nd b	nd b	**	Fruit, Pungent
1.79	2,3-butanedione	0.307 ± 0.006 b	0.367 ± 0.032 b	0.523 ± 0.185 b	1.670 ± 0.816 a	**	Butter, Pastry, Yeast
7.20	2-methyl-3-heptanone	2.083 ± 0.129 a	1.743 ± 0.274 ab	1.480 ± 0.105 b	1.790 ± 0.203 ab	*	Green
7.72	2-heptanone	0.060 ± 0.000 b	nd c	0.093 ± 0.023 a	nd c	**	Blue Cheese, Fruit, Green, Nut, Spice
11.18	Acetoin	0.833 ± 0.038 c	0.870 ± 0.070 c	1.220 ± 0.036 b	5.043 ± 0.348 a	**	Butter, Creamy, Green Pepper
	Total	~5.676	~4.173	~5.776	~12.235		
	Alcohols					*p*-value	
1.31	Ethyl alcohol	8.253 ± 0.626 c	10.236 ± 0.733 b	13.633 ± 1.268 a	4.756 ± 0.366 d	**	Vinous, Pungent
3.12	2-butanol	0.897 ± 0.068 b	0.553 ± 0.040 b	0.800 ± 0.060 b	1.310 ± 0.204 a	**	Alcohol Odor
3.48	1-propanol	0.653 ± 0.150 a	0.817 ± 0.151 a	0.977 ± 0.355 a	0.823 ± 0.117 a	ns	Alcohol, Candy, Pungent
5.02	2-methylpropanol	1.300 ± 0.098 b	0.940 ± 0.040 b	1.177 ± 0.081 b	3.190 ± 0.370 a	**	Apple, Bitter, Cocoa, Wine
5.97	2-pentanol	1.213 ± 0.012 a	1.170 ± 0.010 ab	1.183 ± 0.035 ab	1.126 ± 0.050 b	*	Fusel Oil, Green
6.73	1-butanol	0.207 ± 0.021 a	0.113 ± 0.015 b	0.153 ± 0.038 b	0.150 ± 0.020 b	*	Fruit
8.90	3-methylbutanol	7.957 ± 0.665 b	7.200 ± 0.214 b	8.270 ± 0.503 b	12.400 ± 0.927 a	**	Burnt, Cocoa, Floral, Malt
20.95	2,3-butanediol	0.417 ± 0.159 a	0.353 ± 0.140 a	0.200 ± 0.095 a	0.413 ± 0.134 a	ns	Flavorless
28.99	benzyl alcohol	0.093 ± 0.021 b	0.077 ± 0.015 b	0.210 ± 0.010 a	nd c	**	Boiled Cherries, Moss, Roasted Bread, Rose
30.33	2-phenylethanol	0.807 ± 0.068 ab	0.730 ± 0.020 b	0.740 ± 0.062 b	0.970 ± 0.166 a	*	Fruit, Honey, Lilac, Rose, Wine
	Total	~21.797	~22.189	~27.343	~25.138		
	Esters					*p*-value	
0.48	Ethyl acetate	2.213 ± 0.154 c	10.760 ± 0.226 a	3.786 ± 0.159 b	2.263 ± 0.253 c	**	Aromatic, Brandy, Grape
1.43	Propanoic acid, ethyl ester	nd b	0.183 ± 0.100 a	nd b	nd b	**	Apple, Pineapple, Rum, Strawberry
1.58	Ethyl isobutyrate	0.113 ± 0.012 a	0.163 ± 0.153 a	0.153 ± 0.023 a	0.167 ± 0.040 a	ns	Fruity, Aromatic
1.95	Methyl butanoate	0.107 ± 0.023 c	0.140 ± 0.053 b	0.346 ± 0.065 a	0.330 ± 0.011 a	**	Apple, Banana, Cheese, Ester, Floral
2.60	Isobutyl acetate	0.240 ± 0.170 a	0.210 ± 0.100 a	0.106 ± 0.031 b	0.100 ± 0.026 b	**	Apple, Banana, Floral, Herb
3.21	Butyric acid ethyl ester	4.727 ± 0.163 b	5.613 ± 0.395 a	4.837 ± 0.461 b	2.637 ± 0.152 c	**	Apple, Butter, Cheese, Pineapple, Strawberry
3.65	Ethyl-2-methyl butyrate	nd b	0.063 ± 0.093 ab	0.223 ±0.169 a	0.137 ± 0.007 ab	*	Apple, Ester, Green Apple, Kiwi, Strawberry
4.10	Ethyl isovalerate	0.260 ± 0.010 c	0.313 ± 0.006 bc	0.363 ± 0.031 ab	0.393 ± 0.062 a	**	Apple, Fruit, Pineapple, Sour
5.79	3-methylbutyl acetate	1.237 ± 0.076 a	0.843 ± 0.084 b	0.537 ± 0.042 c	0.450 ± 0.066 c	**	Apple, Banana, Glue, Pear
9.62	Ethyl hexanoate	1.390 ± 0.015 a	1.380 ± 0.332 a	1.583 ± 0.011 a	0.763 ± 0.116 b	**	Apple Peel, Brandy, Overripe Fruit, Pineapple
16.37	Ethyl caprylate	0.113 ± 0.186 a	0.157 ± 0.031 a	0.160 ± 0.056 a	nd b	**	Apricot, Brandy, Fat, Floral, Pineapple
27.31	2-phenylethyl acetate	0.200 ± 0.000 a	0.120 ± 0.000 b	0.027 ± 0.046 c	0.143 ± 0.057 ab	**	Flower, Honey, Rose
29.87	Methyl hexadecanoate	0.173 ± 0.006 a	0.147 ± 0.015 b	0.127 ± 0.006 b	0.150 ± 0.020 ab	*	-
41.43	Methyl octadecanoate	0.070 ± 0.017 b	0.017 ± 0.047 c	nd c	0.137 ± 0.059 a	**	-
	Total	~10.843	~ 20.109	~12.248	~7.67		
	Acids					*p*-value	
16.83	Acetic acid	10.496 ± 0.895 a	12.060 ± 0.755 a	10.576 ± 2.501 a	5.623 ± 2.141 b	**	Acid, Fruit, Pungent, Sour, Vinegar
19.60	Propanoic acid	0.907 ± 0.040 a	0.597 ± 0.021 b	0.570 ± 0.044 b	0.610 ± 0.075 b	**	Fat, Fruit, Pungent, Silage, Soy
20.53	Propanoic acid, 2-methyl-	5.933 ± 0.327 b	3.840 ± 0.242 c	4.700 ± 0.649 bc	8.790 ± 1.405 a	**	Burnt, Butter, Cheese, Sweat
22.28	Butanoic acid	27.800 ± 0.660 a	23.550 ± 0.520 b	23.666 ± 1.341 b	22.280 ± 0.430 c	**	Butter, Cheese, Sour
23.51	Pentanoic acid	6.547 ± 0.461 b	5.523 ± 0.465 b	6.043 ± 0.805 b	9.560 ± 1.508 a	**	Cheese, Pungent
28.32	Hexanoic (caproic) acid	3.680 ± 0.111 a	3.010 ± 0.123 ab	3.226 ± 0.744 ab	2.387 ± 0.611 c	*	Cheese, Oil, Pungent, Sour
33,69	Octanoic (caprylic) acid	4.237 ± 0.021 a	3.197 ± 0.006 b	3.257 ± 0.015 b	2.143 ± 0.021 c	**	Cheese, Fat, Grass, Oil
36.18	Nonanoic acid	nd a	0.040 ± 0.000 a	nd a	nd a	ns	Fat, Green, Sour
42.20	Benzoic acid	0.070 ± 0.062 ab	0.027 ± 0.046 b	0.130 ± 0.017 a	0.090 ± 0.010 ab	*	Pungent, Sour
	Total	~59.670	~51.844	~52.168	~51.033		
	Terpenes					*p*-value	
2.67	*α*-pinene	0.237 ± 0.025 a	0.200 ± 0.020 a	0.273 ± 0.032 a	0.277 ± 0.076 a	ns	Cedarwood, Pine, Sharp
5.20	*β*-pinene	0.075 ± 0.025 a	0.050 ± 0.000 a	0.086 ± 0.005 a	0.070 ± 0.025 a	ns	Pine, Polish, Wood
8.50	*d*-Limonene	0.183 ± 0.059 a	0.097 ± 0.040 a	0.157 ± 0.025 a	0.150 ± 0.053 a	ns	Citrus, Mint
	Total	~0.495	~0.347	~0.516	~0.497		
	Others					*p*-value	
0.15	Hexamethyl- cyclotrisiloxane	0.487 ± 0.083 b	0.337 ± 0.097 b	0.440 ± 0.056 b	0.910 ± 0.155 a	**	-
2.45	Octamethyl-cyclotetrasiloxane	0.210 ± 0.087 a	0.166 ± 0.060 a	0.160 ± 0.036 a	0.193 ± 0.060 a	ns	-
13.98	Dodecamethyl-cyclohexasiloxane	0.160 ± 0.036 a	0.120 ± 0.036 a	0.143 ± 0.012 a	0.137 ± 0.038 a	ns	-
19.39	Tetradecamethyl-cycloheptasiloxane	0.097 ± 0.012 a	0.077 ± 0.012 a	0.090 ± 0.010 a	0.103 ± 0.006 a	ns	-
23.21	Dimethylsilanediol	0.300 ± 0.030 a	0.303 ± 0.137 a	0.237 ± 0.015 a	0.390 ± 0.098 a	ns	-
26.48	Oxime-, methoxy-phenyl-	0.153 ± 0.012 a	0.140 ± 0.030 a	0.133 ± 0.006 a	0.187 ± 0.021 a	ns	-
	Total	~1.407	~1.143	~1.203	~1.920		

Different lower letters between samples in the same line indicate significant differences (* *p* < 0.05; ** *p* < 0.01; ns: not significant; nd: not detected). *** Flavor and Extract Manufacturers Association (FEMA), https://pubchem.ncbi.nlm.nih.gov/compound/ (accessed on 15 April 2026). KS-C: Kemah salt cheese; SS-C: Sivas salt cheese; CS-C: Çankırı salt cheese; LNaS-C: Low-sodium salt cheese.

## Data Availability

The data presented in this study are available from the corresponding authors upon reasonable request.

## Abbreviations

The following abbreviations are used in this manuscript:

PDO	Protected Designation Of Origin
WHO	World Health Organization
KS	Kemah Spring Salt
SS	Sivas Spring Salt
CS	Çankırı Rock Salt
LNaS	Low-Sodium Salt (50% Natural Rock Salt–50% KCl)
FFA	Free Fatty Acids
SCFA	Short-Chain Fatty Acids
MCFA	Medium-Chain Fatty Acids
LCFA	Long-Chain Fatty Acids
SFA	Saturated Fatty Acids
MUFA	Monounsaturated Fatty Acids
PUFA	Polyunsaturated Fatty Acids
TFA	Total Fatty Acids
KS-C	Kemah salt cheese
SS-C	Sivas salt cheese
CS-C	Çankırı salt cheese
LNaS-C	Low-sodium salt cheese
TN	Total Nitrogen
WSN	Water Soluble Content
RI	Ripening Index
